# Castleman disease misdiagnosed as immunoglobulin G4-related disease: a case report

**DOI:** 10.3389/fimmu.2025.1532627

**Published:** 2025-01-28

**Authors:** Na Li, LiFen Xu, Xiaoxia Liu, Pengjia Wu, Jun Liu, Jiashun Zeng

**Affiliations:** ^1^ Department of Rheumatology and Immunology, The Affiliated Hospital of Guizhou Medical University, Guiyang, China; ^2^ Pathology Department, The Affiliated Hospital of Guizhou Medical University, Guiyang, China

**Keywords:** Castleman disease, IgG4-related disease, immune-mediated disorders, lymphadenopathy, accurate diagnosis

## Abstract

Castleman disease (CD) and immunoglobulin G4-related disease (IgG4-RD) are rare systemic immune-mediated disorders that share overlapping clinical features, posing significant challenges in differential diagnosis. Here, we present a case of generalized lymphadenopathy initially misdiagnosed as IgG4-RD, which demonstrated a poor response to hormonal therapy. Subsequent pathological biopsy and immunohistochemical analysis ultimately confirmed the diagnosis of CD. This case underscores the limitations of relying solely on serum IgG4 levels as a diagnostic marker to distinguish CD from IgG4-RD. Comprehensive evaluation, including clinical presentation, organ involvement, serological and pathological findings, as well as therapeutic response, is essential to ensure accurate diagnosis and timely management.

## Introduction

Castleman disease (CD), also known as giant lymph node hyperplasia or angiofollicular lymph node hyperplasia, was first described by Castleman et al. in 1956. CD is classified into unicentric CD (UCD) and multicentric CD (MCD) based on the extent of lymph node and organ involvement. UCD is a benign, localized lymphoid proliferation that is typically curable, while MCD is a systemic lymphoproliferative disorder that often affects multiple organs, leading to dysfunction (1). Certain autoimmune diseases, such as systemic lupus erythematosus and immunoglobulin G4-related disease (IgG4-RD), can mimic MCD by presenting with disseminated lymphadenopathy and “Castleman-like” histopathology, suggesting shared pathophysiologic mechanisms (2). Distinguishing MCD from these mimickers is essential but remains challenging for clinicians.

Herein, we present a case of CD initially misdiagnosed as IgG4-RD, with the aim of providing insights into the distinguishing features of CD and highlighting the importance of timely diagnosis and treatment to improve clinical outcomes.

## Case report

A 36-year-old male presented with “seven years of weakness with enlarged lymph nodes and six months of back pain.” In August 2015, the patient experienced generalized weakness and multiple enlarged lymph nodes (largest approximately 3 × 2 cm) predominantly in the preauricular, retroauricular, anterior and posterior cervical, submandibular, supratrochlear, and inguinal regions. He was treated with cephalosporins at a local hospital without symptom improvement.

In May 2016, persistent weakness, lymph node enlargement, and night sweats prompted further evaluation at another hospital. Serum globulin was 40+ g/L, and a right retroauricular lymph node biopsy revealed “reactive lymph node hyperplasia, ruling out non-Hodgkin’s lymphoma required.” Immunohistochemistry showed CD138+, IgG4 (+, >200/HPF), and Igκ++. The diagnosis was reported as “immune-related lymphoproliferative lesions, IgG4-RD?” After receiving 50 mg/day of prednisone acetate, the patient self-discharged due to lack of symptom improvement.

**Figure 1:**
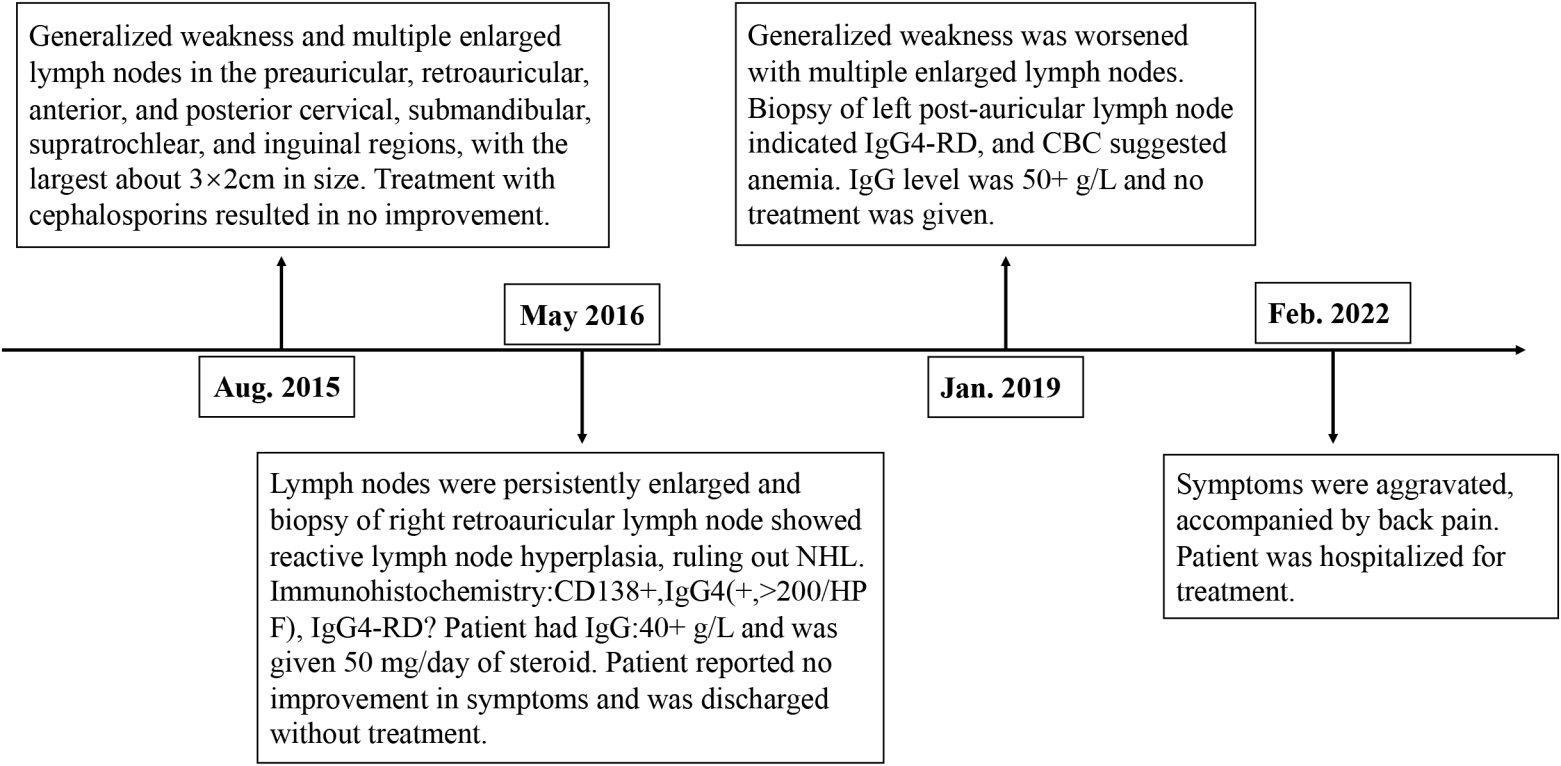
Timeline of patient diagnosis and treatment.

In January 2019, the patient reported worsening weakness and generalized lymph node enlargement. A left retroauricular lymph node biopsy suggested IgG4-RD, with serum globulin levels elevated to 50+ g/L. No treatment was initiated. In February 2022, the patient’s symptoms further worsened with back pain and progressive lymph node enlargement. He sought care at our outpatient clinic, was diagnosed with “IgG4-RD,” and subsequently admitted to our department (**Figure 1**).

Since disease onset, the patient denied fever, dysphagia, rash, malar erythema, joint pain, Raynaud’s phenomenon, chest tightness, or chest pain. His mental well-being was acceptable despite poor diet, sleep quality, and nocturnal enuresis. He reported normal bowel movements and a weight loss of approximately 5 kg. Twenty years ago, he was diagnosed with vitiligo, currently presenting with well-defined milky-white patches on the neck, dorsum of the hands, forearms, and head, covering less than 5% of the body surface area, without specific treatment. He denied systemic diseases such as hypertension, diabetes, or coronary heart disease. Personal, family, and marital history were unremarkable.

Upon admission, the patient had stable vital signs but appeared chronically ill and anemic. Skin examination revealed no rash, but scattered pigmentation loss was noted on the extremities. Multiple enlarged lymph nodes were palpated in the preauricular, retroauricular, anterior and posterior cervical, submandibular, buccal, and groin regions, with the largest measuring approximately 3 × 2 cm. These lymph nodes were firm, mobile, non-tender, and had distinct margins. The spleen was palpable 4 cm below the rib cage, soft, and non-tender. Cardiopulmonary and joint examinations revealed no abnormalities, and muscle strength and tone were normal in all extremities, with no edema observed in the lower limbs.

Laboratory tests showed a WBC count of 6.12 × 10⁹/L, hemoglobin of 64 g/L, and platelets of 349 × 10⁹/L. Blood chemistry revealed albumin 22 g/L, globulin 70.6 g/L, ALT 14.8 U/L, AST 21.4 U/L, total bilirubin 12.1 µmol/L, creatinine 107 µmol/L, and an eGFR of 41.05 mL/min/1.73 m². Urinalysis showed protein (2+) and 24-hour urinary protein of 1979.08 mg/24 h with a total urine volume of 2500 mL. IL-6 was elevated at 179.6 pg/mL, CRP at 110.27 mg/L, and ESR was 19 mm/h. ANA showed a titer of 1:320 with a cytoplasmic speckled pattern. Immunoglobulin levels were IgG 49.9 g/L, IgA 3.98 g/L, and IgM 0.472 g/L, with complement C3 at 1.21 g/L and C4 at 0.188 g/L. IgG4 was markedly elevated at 9869.6 mg/L, and p-ANCA was positive. Screening for Coomb’s test, anticardiolipin antibody, rheumatoid factor, anti-CCP antibody, immunofixation electrophoresis, tumor markers, hepatitis B, Human herpesvirus 8, Cytomegalovirus, Epstein-Barr virus, and infectious diseases was negative (**Table 1**). Protein electrophoresis showed no detectable M protein.

**Table 1:** Patient serology.

Serology test	Result	Normal range
White blood cell	6.12×10^9^/L	3.5-9.5×10^9^/L
Hemoglobin	64 g/L	130-175g/L
Patelets	349 × 10⁹/L	125-350 × 10⁹/L
Albumin	22 g/L	40-55g/L
Globulin	70.6 g/L	20-40g/L
Alanine aminotransferase	14.8 U/L	9-50 U/L
Aspartate aminotransferase	21.4 U/L	15-40 U/L
Total bilirubin	12.1 µmol/L	<23 µmol/L
Creatinine	107 µmol/L	57-97 µmol/L
Glomerular filtration rate	41.05 mL/min/1.73 m²	>90 mL/min/1.73 m²
Interleukin-6	179.6 pg/mL	0-7 pg/mL
C-reactive protein	110.27 mg/L	<5mg/L
Blood sedimentation	19 mm/h	0-21mm/h
Immunoglobulin G	49.9 g/L	8.6-17.4 g/L
Immunoglobulin A	3.98 g/L	1-4.2 g/L
Immunoglobulin M	0.472 g/L	0.3-2.2 g/L
Complement C3	1.21 g/L	0.7-1.4 g/L
Complement C4	0.188 g/L	0.1-0.4 g/L
Immunoglobulin G4	9869.6 mg/L	≤2000 mg/L
Total immunoglobulin E	94 IU/mL	0-100 IU/mL
Urinary protein	++	–
24-hour urinary protein	1979.08 mg/24 h	<140 mg/24h
Antineutrophil cytoplasmic antibody was perinuclear	p-ANCA(+)	–

Chest and abdominal CT revealed bilateral lung infections, bronchiolitis, mild bronchiectasis in the right middle and both lower lobes, mediastinal and retroperitoneal lymphadenopathy, restrictive emphysema in the upper lobes, hepatosplenomegaly, calcified foci in the left liver lobe, and small calculi in the right kidney.

**Figure 2:**
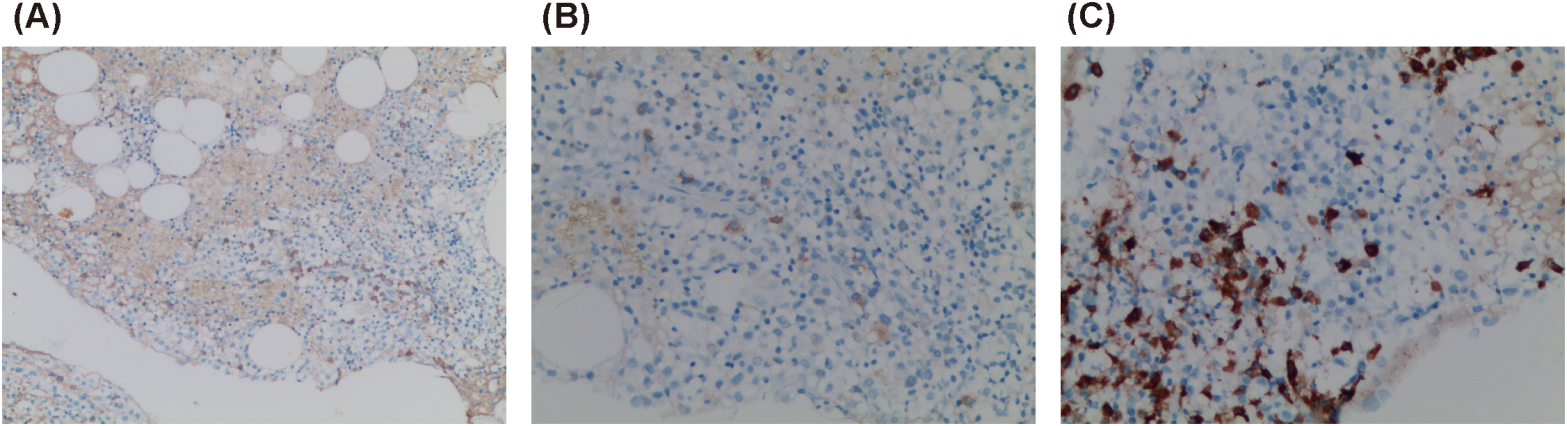
Bone marrow biopsy. **(A)** IgG (+) (×100); **(B)** IgG4 (+) (×200); **(C)** CD79α (+) (×200).

A standard bone marrow smear showed proliferative anemia with iron depletion and rare progenitor and immature lymphoid cells, suggesting the need for clinical correlation to rule out lymphocyte clonal disease. Bone marrow biopsy revealed hypoproliferative marrow with small lymphocyte hyperplasia. Immunohistochemistry demonstrated hyperproliferation of mature lymphocytes and plasma cells, with CD138 (+, 5%–10%), CD38 (+, approximately 5% in hotspot areas), CD79α (+, 5%–10%), CD56 (+, few), CD3 (+, few), CD4 (+, few), CD8 (+, few), multiple myeloma oncogene-1 (-), IgA (+, individual), IgG (+, few), IgG4 (+, individual), IgM (+, individual), Lambda (+, few), Kappa (+, few), and Ki-67 (+, approximately 20%) (**Figure 2**).

Puncture biopsy of the left cervical lymph node revealed proliferative lymphoid lesions characterized by partial closure of the subcapsular sinus, a few follicles in both the subcapsular region and the center of the lymph node, atrophic germinal centers (GCs) with vascular hyperplasia in some follicles, and proliferation and patchy distribution of a significant number of mature plasma cells within the interfollicular space. Vascular hyperplasia penetrating the GCs, proliferation of histiocytes in the local lymph sinus, and erythrophagocytosis were also observed. Immunohistochemical staining showed CD79α (+, B cells), CD138 (+, plasma cells), CD38 (+), multiple myeloma oncogene-1 (+, plasma cells), CD3 and CD5 (partial +), CD56 (+, scattered), CD10 (+ in GCs), CD21 (+, showing shrinkage and disruption of the FDC network), Kappa and Lambda (partial +), CD34 (+, showing vascular hyperplasia penetrating GCs), IgG4 (>100/HPF), IgG (partial +), IgG4+ cells less than 40% of IgG+ cells, IgM (+, individual cells), IgA (few +), IgD (individual +), and IgG4+/IgG+ approximately 40%. The proliferation index Ki-67 was approximately 60% in the GCs, 40% in parts of the GCs, and 10% outside the GCs.

**Figure 3:**
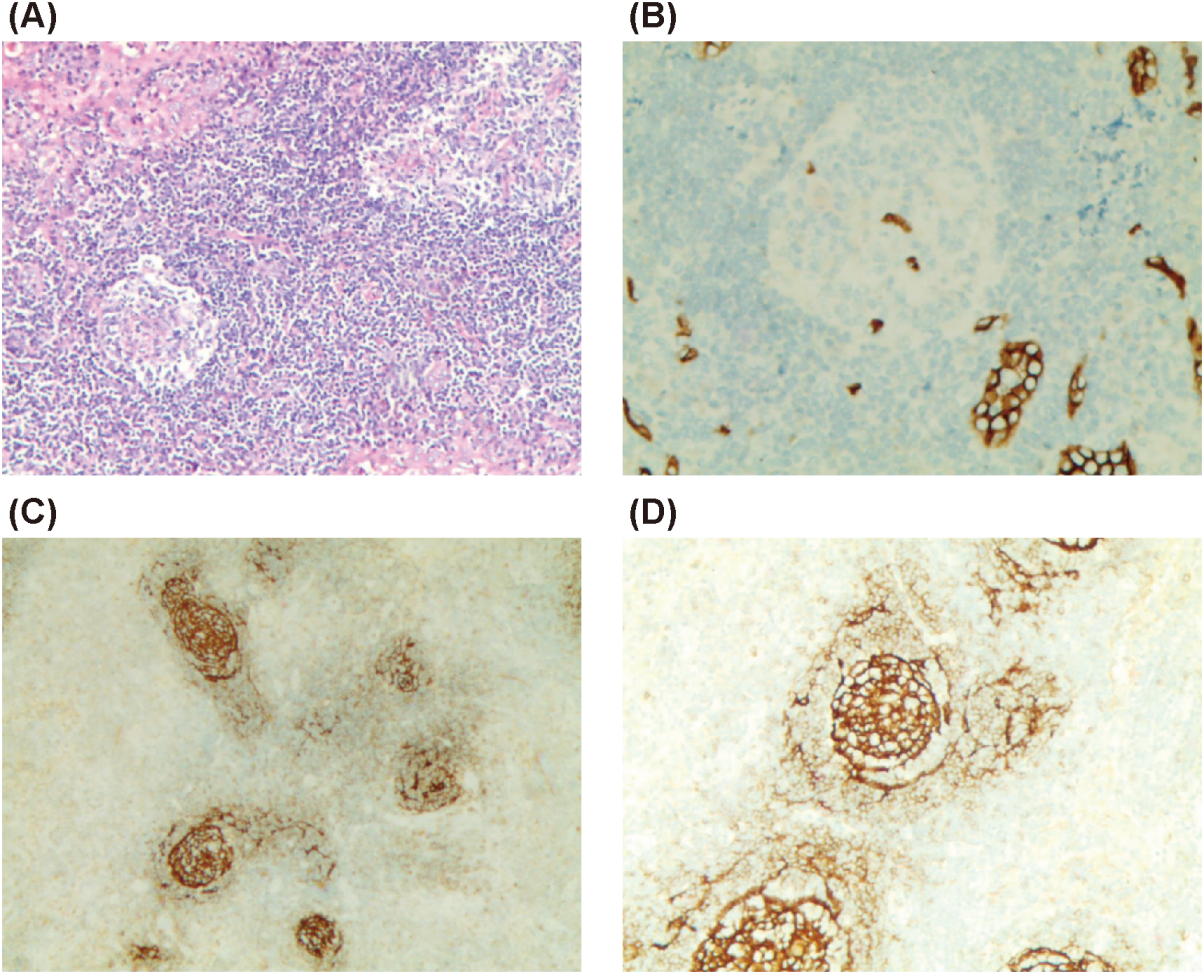
Biopsy of left cervical lymph node. **(A)** Partial closure of subcapsular sinus, few follicles in the subcapsular region and the center of the lymph node, and atrophy of some GCs under low magnification (×100); **(B)** CD34 (+, showing vascular hyperplasia penetrating GCs) (×200); **(C)** CD21 (+, showing shrinkage and destruction of the FDC network) (×100); **(D)** CD21 (+) (×200).

These findings, along with the observed lymphadenopathy, were consistent with a diagnosis of Castleman disease (CD) (**Figure 3**).

Renal biopsy pathology revealed 21 glomeruli, among which two showed segmental sclerosis. Globular and ischemic sclerosis accounted for approximately 33% of all glomeruli. The remaining glomeruli exhibited mild diffuse hyperplasia of mesangial cells and mesangial stroma, with moderate focal segmental aggravation and endothelial cell hyperplasia. Additional findings included glomerular basement membrane wrinkling, extensive vacuolar degeneration, segmental nailing, tram-tracking, focal tubular atrophy, epithelial vacuolation with granular deformation, and interstitial lymphocyte and monocyte infiltration accompanied by fibrosis.

**Figure 4:**
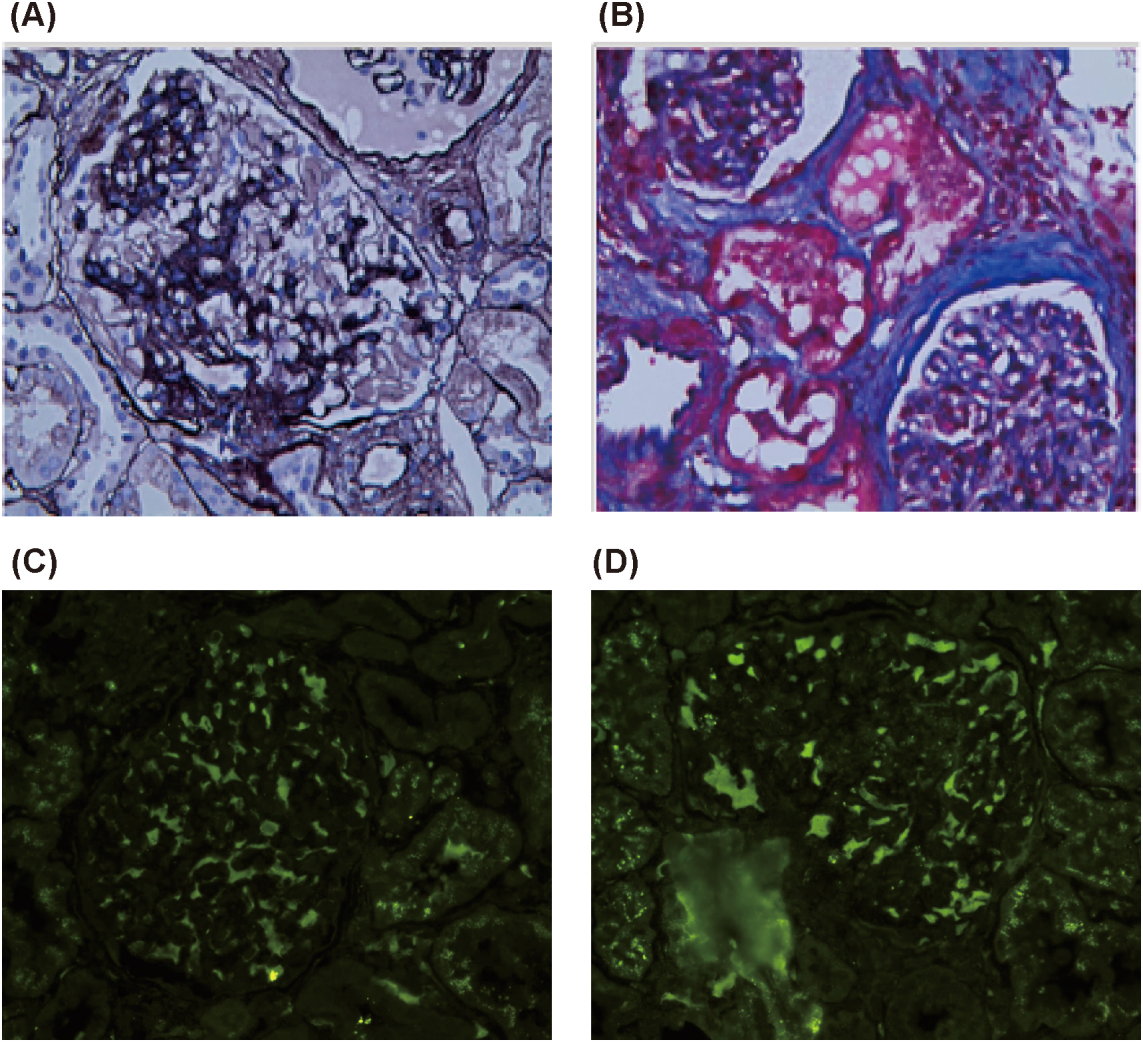
Renal puncture biopsy. **(A, B)** Hexamine silver and Masson staining showing immune complex deposition and thickened basement membrane, respectively; **(C, D)** IgA1 (++++) (×400). Findings are consistent with focal proliferative sclerosing IgA nephropathy with membranous nephropathy.

Immunofluorescence showed IgG+, IgA+, IgM+, C3±, C1q±, IgG1±, IgG2-, IgG3-, and IgG4-. These findings suggested focal proliferative sclerosing IgA nephropathy (M1E1S1T1) with features of membranous nephropathy (**Figure 4**).

Based on the patient’s clinical manifestations, laboratory results, and imaging findings, infectious diseases, solid tumors, and autoimmune diseases were ruled out. The patient was ultimately diagnosed with multicentric Castleman disease (MCD) and started on 600 mg of siltuximab every three weeks. Two months after treatment, a repeat physical examination revealed a reduction in superficial lymph node enlargement. Laboratory tests showed improved liver and renal function: albumin 32.8 g/L, globulin 48.66 g/L, alanine aminotransferase 14.4 U/L, aspartate aminotransferase 13.7 U/L, total bilirubin 6.3 µmol/L, creatinine 93 µmol/L, and an estimated glomerular filtration rate (eGFR) of 50.48 mL/min/1.73 m². Urinalysis was negative for protein, with IgG 30.8 g/L and β2-microglobulin 4.81 mg/L.

## Discussion

The etiology and pathogenesis of Castleman disease (CD) remain unclear. Recent studies suggest that its pathogenesis may involve excessive IL-6 production, human immunodeficiency virus (HIV) infection, human herpesvirus-8 (HHV-8) infection, chronic inflammation, immune regulatory disorders, and epidermal growth factor receptor (EGFR) overexpression. Multicentric Castleman disease (MCD) is classified into HHV-8-positive MCD and HHV-8-negative MCD, also known as idiopathic MCD (iMCD), based on HHV-8 infection status (3). iMCD accounts for approximately one-third to one-half of all MCD cases and has a higher prevalence in the Chinese population. It can occur at any age, with no significant gender difference in incidence. iMCD is associated with poor outcomes, with a five-year survival rate ranging from 55% to 77% (4).

Patients with iMCD exhibit varying degrees of systemic inflammation, polyclonal lymphocyte proliferation, and a wide range of symptoms driven by cytokine storm. These include fever, night sweats, lymphadenopathy, ascites, hepatosplenomegaly, renal impairment, elevated CRP, hypoalbuminemia, and anemia. In 2017, the Castleman Disease Collaborative Network established diagnostic criteria for iMCD, comprising two major criteria (both required): histopathologic lymph node features (e.g., regressed/atrophic germinal centers, increased vascularity, polytypic plasmacytosis) and lymphadenopathy in at least two lymph node stations. Additionally, at least two of 11 minor criteria are required, including at least one laboratory criterion. Minor criteria include elevated ESR or CRP, anemia, thrombocytopenia, hypoalbuminemia, elevated serum IgG, renal dysfunction, splenomegaly or hepatomegaly, fluid accumulation, cherry hemangiomatosis or violaceous papules, lymphocytic interstitial pneumonitis, and constitutional symptoms such as fever, night sweats, or weight loss. The diagnosis also requires exclusion of infections, neoplasms, and autoimmune disorders (3).

IgG4-related disease (IgG4-RD) is a systemic immune-mediated fibroinflammatory disorder characterized by elevated serum IgG4 levels. It can affect multiple organs and is often misdiagnosed as a malignancy, infection, or other immune-mediated conditions. IgG4-RD predominantly occurs in middle-aged and older individuals, with a male-to-female ratio of approximately 8:3 (5). The exact etiology and pathogenesis of IgG4-RD remain unclear, but genetic predisposition, environmental factors, autoantibodies, and dysregulation of both innate and adaptive immunity are thought to contribute to its development. IgG4-RD presents with a wide range of clinical manifestations, including lymphadenopathy, pancreatic and bile duct impairment, salivary gland damage, renal lesions, and retroperitoneal fibrosis. Its hallmark histopathologic features include IgG4+ plasma cell infiltration, storiform fibrosis, and obliterative phlebitis.

The current diagnostic criteria for IgG4-RD include the 2020 revised comprehensive diagnostic criteria and the 2019 American College of Rheumatology (ACR)/European League Against Rheumatism (EULAR) classification criteria (6, 7). Compared to the 2011 criteria, the 2019 criteria place greater emphasis on ruling out alternative diseases and quantifying infiltrating lymphoid and IgG4+ plasma cells in affected organs, underscoring the need for a comprehensive diagnostic approach. CD and IgG4-RD share overlapping clinical features, such as lymphadenopathy and elevated serum IgG4 levels, making differentiation between the two conditions challenging. In this case, the patient presented with generalized lymphadenopathy and renal lesions, with pathological immunohistochemistry of the right auricular lymph node biopsy revealing over 200 IgG4-positive plasma cells under high magnification. Additional findings included hyperglobulinemia, markedly elevated serum IgG4 levels, and increased IL-6 and CRP levels. No evidence of infections or neoplasms was detected, appearing to meet the 2020 IgG4-RD diagnostic criteria.

To further clarify the diagnosis, a left cervical lymph node biopsy was performed. Pathological examination showed atrophic germinal centers (GCs) with vascular proliferation, extensive mature plasma cell hyperplasia in the interfollicular spaces, and vascular hyperplasia penetrating the GCs. The IgG4+/IgG+ plasma cell ratio was approximately 40%. These findings were consistent with CD, leading to a final diagnosis of idiopathic multicentric Castleman disease (iMCD) involving the lymph nodes, liver, spleen, blood system, and kidneys. The patient’s condition improved rapidly with siltuximab treatment, further supporting the diagnosis of CD.

CD and IgG4-RD share overlapping clinical and laboratory features, but their distinct pathogenesis requires different treatment approaches. Nonetheless, these two diseases can be differentiated through several approaches (8, 9): (1) Clinical manifestations: IgG4-RD more often involves glands, such as salivary glands, lacrimal glands, or the pancreas, whereas MCD results in more prominent lymph node enlargement and is often accompanied by constitutional symptoms including fever, weakness, and weight loss. (2) Serological biomarkers: Patients with MCD have significantly elevated CRP, IL-6, ESR, IgG, IgA, IgM, and IgE levels but markedly decreased hemoglobin and albumin levels compared with IgG4-RD. In contrast, patients with IgG4-RD tend to have normal levels of IL-6 and CRP but increased eosinophil counts and IgG4/IgG ratios, with some exhibiting decreased C3 and C4 levels. (3) Pathological features: IgG4+ plasma cell infiltration is observed in both diseases. However, patients with MCD do not have eosinophilic infiltration or laminar fibrosis, and their IgG4+/IgG+ plasma cell ratio is less than 40%. This contrasts with the >40% IgG4+/IgG+ ratio observed in the tissues of patients with IgG4-RD. (4) Response to glucocorticosteroids: Patients with IgG4-RD are highly responsive to initial glucocorticosteroids, while patients with MCD are less responsive.

Renal involvement in CD is uncommon, and little is known about the role of the kidneys in CD progression. Renal pathology in iMCD is complex and heterogeneous. A retrospective study of 73 patients with iMCD who underwent renal biopsy found that most renal lesions were not specific to iMCD but were due to immune-mediated secondary renal damage, with amyloidosis being the most frequently reported lesion in the literature (10). However, a Chinese cohort study showed that renal involvement was predominantly observed in MCD, with thrombotic microangiopathy-like lesions being the most common. Furthermore, renal involvement did not affect the patients’ life expectancy (11). The renal pathology findings of our patient also lacked iMCD-specific features, which were assessed as immune-mediated secondary IgA nephropathy with membranous nephropathy. These renal pathologic features are rarely reported in the literature. Some scholars have suggested that the mechanism of renal injury may be related to the abnormal increase in IL-6 associated with immune dysfunction (12). Additionally, the excessive secretion of IL-6 by the affected lymph nodes can induce B cell maturation, stimulate the synthesis of immunoglobulins in the acute phase, and promote the proliferation of mesangial cells and stroma, thereby damaging the kidneys. It has also been found that patients with CD exhibit hypersensitive immune responses to antigens, resulting in the deposition of immune complexes in the glomerular basement membrane and the mesangial zone, thereby contributing to kidney damage (13).

## Conclusion

CD and IgG4-RD share similar clinical features, making differential diagnosis challenging. Serum IgG4 levels alone are unreliable as a diagnostic marker, whereas IL-6 levels may serve as a key differentiator between these conditions. Accurate diagnosis relies on prompt biopsy of enlarged lymph nodes and affected organs, combined with comprehensive histopathological and immunohistochemical analyses.

## Data Availability

The original contributions presented in the study are included in the article/supplementary material. Further inquiries can be directed to the corresponding author.
